# Identification of Pathways in Liver Repair Potentially Targeted by Secretory Proteins from Human Mesenchymal Stem Cells

**DOI:** 10.3390/ijms17071099

**Published:** 2016-07-09

**Authors:** Sandra Winkler, Madlen Hempel, Sandra Brückner, Hans-Michael Tautenhahn, Roland Kaufmann, Bruno Christ

**Affiliations:** 1 Applied Molecular Hepatology Laboratory, Department of Visceral, Transplantation, Thoracic and Vascular Surgery, University Hospital of Leipzig, Liebigstraße 21, 04103 Leipzig, Germany; sandra.pelz@medizin.uni-leipzig.de (S.W.); madlen.hempel@medizin.uni-leipzig.de (M.H.); sandra.brueckner@medizin.uni-leipzig.de (S.B.); hans-michael.tautenhahn@medizin.uni-leipzig.de (H.-M.T.); 2 Department of General, Visceral and Vascular Surgery, Jena University Hospital, Erlanger Allee 101, 07747 Jena, Germany; roland.kaufmann@med.uni-jena.de

**Keywords:** mesenchymal stem cells, secretome, cytokines, chemokines, liver regeneration, hepatocytic differentiation, bone marrow, adipose tissue

## Abstract

Background: The beneficial impact of mesenchymal stem cells (MSC) on both acute and chronic liver diseases has been confirmed, although the molecular mechanisms behind it remain elusive. We aim to identify factors secreted by undifferentiated and hepatocytic differentiated MSC in vitro in order to delineate liver repair pathways potentially targeted by MSC. Methods: Secreted factors were determined by protein arrays and related pathways identified by biomathematical analyses. Results: MSC from adipose tissue and bone marrow expressed a similar pattern of surface markers. After hepatocytic differentiation, CD54 (intercellular adhesion molecule 1, ICAM-1) increased and CD166 (activated leukocyte cell adhesion molecule, ALCAM) decreased. MSC secreted different factors before and after differentiation. These comprised cytokines involved in innate immunity and growth factors regulating liver regeneration. Pathway analysis revealed cytokine-cytokine receptor interactions, chemokine signalling pathways, the complement and coagulation cascades as well as the Januskinase-signal transducers and activators of transcription (JAK-STAT) and nucleotide-binding oligomerization domain-like receptor (NOD-like receptor) signalling pathways as relevant networks. Relationships to transforming growth factor β (TGF-β) and hypoxia-inducible factor 1-α (HIF1-α) signalling seemed also relevant. Conclusion: MSC secreted proteins, which differed depending on cell source and degree of differentiation. The factors might address inflammatory and growth factor pathways as well as chemo-attraction and innate immunity. Since these are prone to dysregulation in most liver diseases, MSC release hepatotropic factors, potentially supporting liver regeneration.

## 1. Introduction

The biological features of mesenchymal stem cells (MSC) make them feasible candidates for cellular therapy for a variety of diseases, e.g., acute kidney injury, brain repair after stroke, colitis [1,2,3,4,5,6], and acute and chronic liver diseases [7,8,9,10,11,12,13,14]. Depending on the therapeutic goal, MSC might be used as undifferentiated cells to provide regenerative support by paracrine actions or after hepatocytic differentiation to provide metabolic capacity, or to bridge the patient to liver transplantation [15]. Currently, a series of clinical phase-I trials using MSC as therapy option to treat liver diseases are in progress [16,17], albeit the molecular mechanisms of the stem cells’ impact remain mostly elusive. At low rates, transplanted MSC engrafted into the host liver and adopted the full hepatocyte phenotype [18,19]. In recent times, however, it has been shown that the beneficial effect of MSC is often mediated by transient, paracrine mechanisms comprising the secretion of soluble factors by the MSC without requiring hepatic engraftment [7,20,21,22,23,24]. If this is the principle of action, then the question arises whether MSC derived from different tissue sources display the same paracrine pattern of secreted factors. In particular, differences in cytokines, chemokines and growth factors involved in hepatocyte differentiation and growth would of course have significant therapeutic impact on liver repair and regeneration. Since cytokines and chemokines mediate both inflammatory and anti-inflammatory responses, it is from a clinical point of view relevant to know, whether a given disease imprints the panel of proteins secreted by the MSC, which might then behave differently under different disease conditions [25,26,27]. In the liver, paracrine and also endocrine factors play an important role in cell and tissue homeostasis. For example, the cytokine interleukin 6 (IL-6) secreted by Kupffer cells is the prominent cytokine initiating the acute phase reaction as the first line of defence against trauma, tissue damage or neoplastic growth, and together with tumour necrosis factor α (TNFα) is the priming factor initiating liver regeneration after damage like for example after partial hepatectomy [28,29]. A row of MSC-derived factors with pleiotropic actions might also potentially foster liver regeneration via multiple pathways like TGFα promoting vascularization and mitogenesis [29,30], or angiogenic factors like vascular endothelial growth factor (VEGF) and angiopoietins 1 and 2 [30]. Conversely, thrombospondin-1 induces apoptosis and antagonizes VEGF by activating the c-Jun N-terminal kinase (JNK) pathway via binding to the scavenger receptor CD36 [31], thus contributing to tissue remodelling during liver regeneration. Furthermore, morphogenic pathways in the liver are impacted by MSC-borne factors like the Wnt pathway via its inhibitor Dickkopf-1, which is crucial for metabolic imprinting of hepatocytes along the sinusoids, and thus for functional homeostasis during tissue regeneration [32]. Besides their capacity to support tissue homeostasis and function, MSC-derived molecules attenuate inflammatory diseases, like TGF-β1 alleviating experimental colitis [6], or the anti-inflammatory cytokines IL-4 and IL-10 inducing polarisation of macrophages into the ”non-inflammatory” M2 phenotype, thus attenuating inflammation in non-alcoholic steatohepatitis (NASH) [25]. Eventually, TGF-β, which does not only display beneficial but also deleterious actions in the liver like initiation of fibrosis by the activation of hepatic stellate cells, is involved in multiple pathways comprising the JAK-STAT, JNK and mitogen-activated protein kinases (MAPK) pathways [33,34] and their crosstalk with cyto- and chemokine pathways mediated by fibroblast growth factors (FGFs) [35], CC chemokine ligands (CCLs) [36], interleukines (ILs) [37] and brain-derived neurotrophic factor (BDNF) [38]. Therefore, it is of ultimate importance before clinical application of MSC to characterise their potential mode of action in respect of their paracrine response to a given liver disease. In this study, we identified cytokine profiles of undifferentiated and hepatocytic differentiated MSC from different tissue origins with the aim to unravel signalling pathways delineating their potential biological effects in vivo. We reasoned that MSC application might target a widespread pattern of biological events in the liver, which may contribute to amelioration of both acute and chronic liver diseases.

## 2. Results

### 2.1. Phenotypic Characteristics

**Figure 1:**
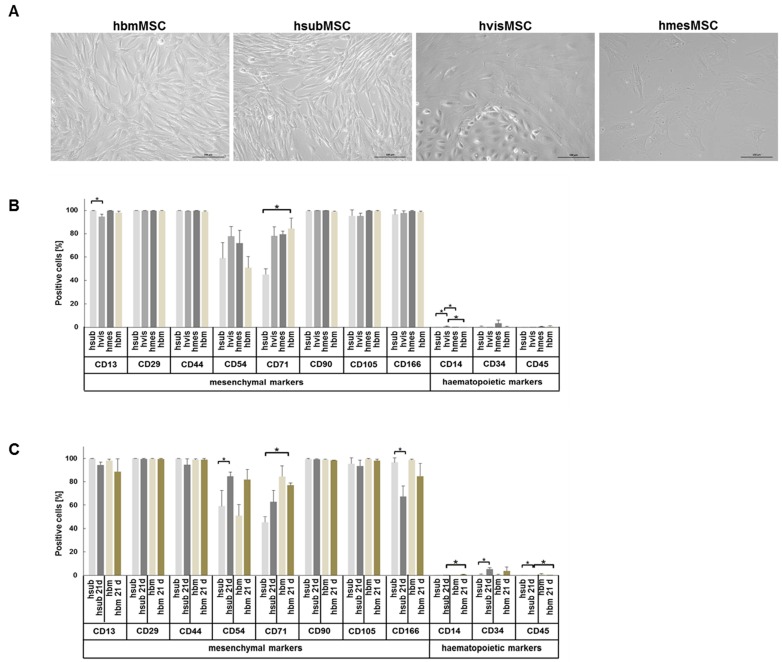
Phenotypic features of mesenchymal stem cells (MSC) from different tissue sources. In (**A**), the morphology of undifferentiated MSC derived from human bone marrow (hbm) and subcutaneous (hsub), visceral (hvis) and mesenteric (hmes) adipose tissue is shown (scale bar: 100 µm). To reach near confluent growth (80%–90%), hbm-, hsub-, and hvis-MSC grew in about 8 days, while hmesMSC needed more than 14 days of culture. (Scale bar: 100 µm); The mesenchymal and hematopoietic surface marker profile (**B**) of undifferentiated MSC derived from subcutaneous (hsub), visceral (hvis), mesenteric (hmes) adipose tissue and bone marrow (hbm) displayed only marginal quantitative differences; After hepatocytic differentiation (**C**) of hsubMSC and hbmMSC, the expression of CD54 increased while that of CD166 decreased significantly (* *p* < 0.05; mean values from three to five independent analyses using cells from different donors each).

As a typical feature of MSC, all subpopulations studied showed adherence to plastic culture surfaces and a spindle-shaped morphology with the exception of visceral adipose tissue-derived MSC, which contained in addition a contaminating subpopulation of untypically rounded cells. While bone marrow and visceral as well as subcutaneous adipose tissue-derived MSC reached confluent growth after about 8 days, mesenteric adipose tissue-derived MSC grew confluent after about 14 days, *bona fide* indicating a lower proliferative capacity (Figure 1A).

The expression of surface marker proteins was determined on all subpopulations of MSC. Yet, due to the ease of availability, only hsubMSC and hbmMSC were further characterized in terms of surface markers and functional features before and after hepatocytic differentiation. Undifferentiated human MSC from either tissue under investigation expressed the mesenchymal surface marker panel comprising CD13, CD29, CD44, CD90, CD105 and CD166 to nearly 100%. Fewer cells expressed CD54 and CD71 and all were virtually negative for the hematopoietic markers CD14, CD34 and CD45. Albeit significant, differences in the expression of CD13 and CD14 were marginal and thus negligible, while the substantial difference in the expression of CD71 between hsubMSC and hbmMSC might be of functional relevance (Figure 1B).

Comparing undifferentiated and hepatocytic differentiated MSC, the expression of CD54 increased and that of CD166 decreased significantly on hsubMSC after differentiation. Although not significant, hbmMSC showed the same trend. Notably, the expression of the hematopoietic marker CD34 increased significantly up to 5.4% after differentiation of hsubMSC (Figure 1C).

### 2.2. Identification of Hepatotropic Factors Secreted by Mesenchymal Stem Cells (MSC)

**Figure 2:**
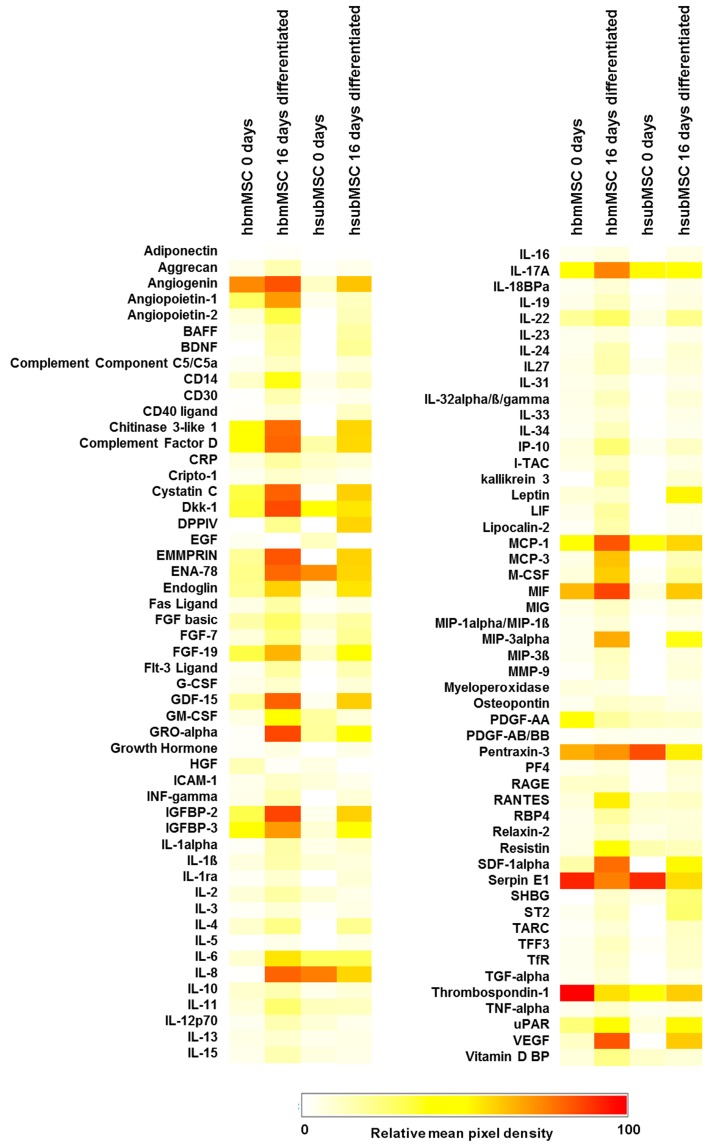
Heatmap of secretory protein abundance of undifferentiated (0 day) and differentiated (16 day) hbmMSC and hsubMSC. The heatmap was created by setting the maximal pixel intensity of the reference spots on the array arbitrarily to 100 (red colour), to which the abundance of all other analytes is relative. Minimal abundance (0) is encoded by white, mean abundance (50) by yellow colouring. Pixel densities shown were calculated as means from three independent experiments with MSC from different donors each.

The analyses of the proteome profiler experiments were graphically summarised in the heatmap shown in Figure 2. Quantitative and qualitative differences were obvious between hbmMSC and hsubMSC, both undifferentiated and after hepatocytic differentiation.

**Figure 3:**
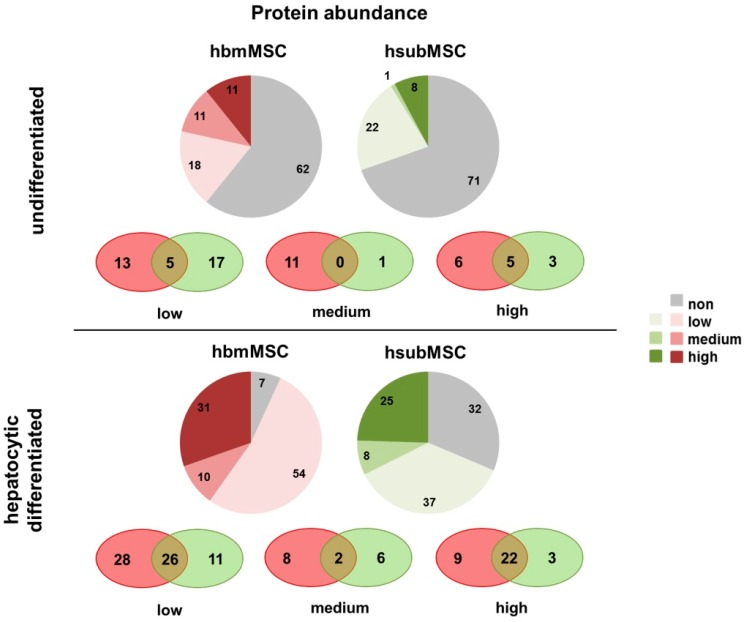
Graphical illustration of proteins secreted by undifferentiated (**top**) and differentiated (**bottom**) hbmMSC (**red**) and hsubMSC (**green**). The pie charts represent the number of proteins arbitrarily classified as low, medium and high secretion. The number of proteins not detected is shown in grey. Proteins secreted both by hbmMSC and hsubMSC are shown as the intersection of proteins secreted at low, medium and high abundance. Estimates were deduced from three independent experiments using MSC from three different donors each.

Using an arbitrary classification, abundance of individual proteins was estimated at low, medium and high secretion (epidermal growth factor (EGF) and hepatocyte growth factor (HGF) were not considered, because both were components of the differentiation media). Protein abundance was different in undifferentiated hbmMSC and hsubMSC. While in media of hbmMSC 40 proteins (18 low, 11 medium, 11 high) were verified, hsubMSC exhibited 31 secreted proteins (22 low, 1 medium, 8 high), part of them overlapping in both as shown in the intersection presentation (Figure 3, top). Both MSC populations secreted IL-17A, monocyte chemotactic protein 1 (MCP-1), Pentraxin-3, SerpinE1 and Thrombospondin-1 in high abundance. IL-8 was highly abundant in supernatants of hsubMSC and not found in supernatants of hbmMSC (Tables S1 and S2).

In general, abundance of most proteins increased after hepatocytic differentiation (Figure 3, bottom). 95 proteins (54 low, 10 medium, 31 high) were secreted by differentiated hbmMSC, and 70 (37 low, 8 medium, 25 high) by differentiated hsubMSC, 50 of which were found in supernatants of both (intersections in Figure 3, bottom).

**Figure 4:**
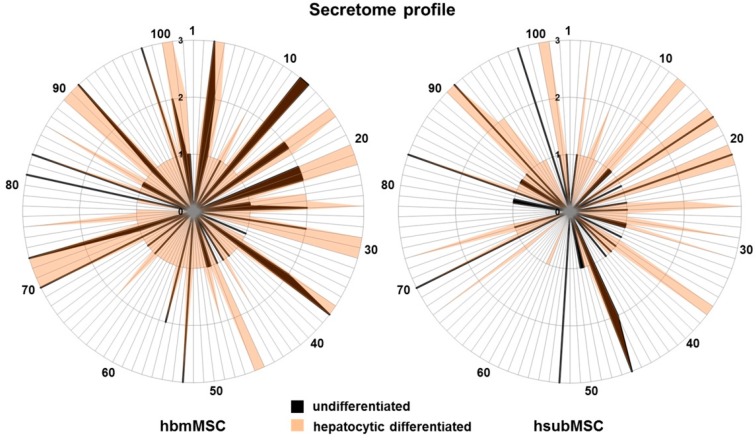
Graphical nets of proteins secreted by undifferentiated (**black** lines) and differentiated (**orange** lines) hbmMSC (**left**) and hsubMSC (**right**) in low (1), medium (2) and high (3) abundance as taken from results shown in Figure 3. Individual proteins are numbered consecutively from 1 to 102 as shown at the edge of the radii. A comprehensive list of numbers and corresponding proteins is given in Table S5.

Besides factors already secreted in high abundance in undifferentiated MSC, hepatocytic differentiation contributed a significant number of additional factors. Lists of individual proteins secreted in low, medium or high abundance by differentiated MSC are summarised in Tables S3 and S4. In order to gain a comprehensive view of qualitative and semi-quantitative changes before and after hepatocytic differentiation, graphical nets were designed, showing that secretory profiles of hsubMSC and hbmMSC were qualitatively and quantitatively different from each other both, before and after differentiation, albeit overlapping to a certain extent (Figure 4).

### 2.3. Identification of Pathways and Networks Affected by Factors Secreted by MSC

**Figure 5:**
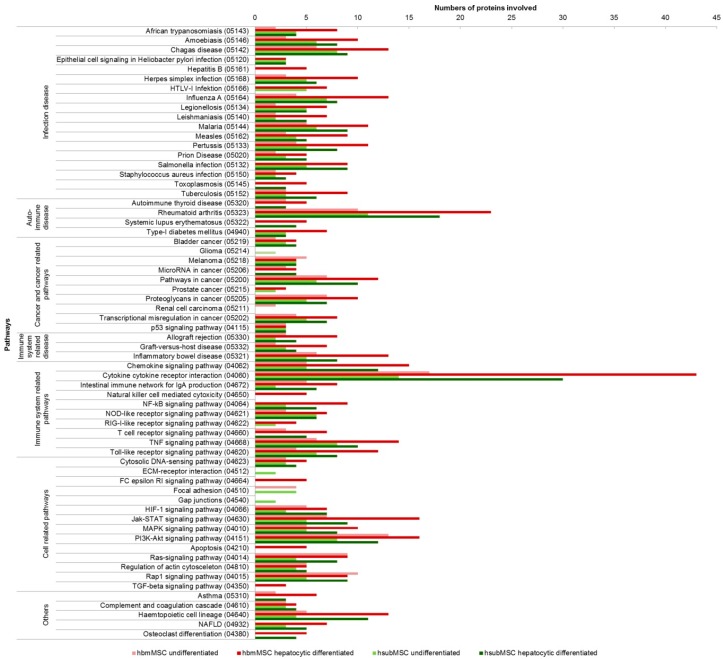
Pathway analysis of proteins secreted by hbmMSC (**red**) and hsubMSC (**green**) before (**light** colours) and after (**dark** colours) hepatocytic differentiation. Pathways were identified using the Search Tool for the Retrieval of Interacting Genes/Proteins (STRING) database (pathway identifiers given in parentheses) and clustered into main disease- or cellular processes-related pathways (*Y*-axis). The number of proteins involved in each single pathway is depicted on the *X*-axis. All pathways shown were significant at the *p* < 0.05 level as calculated from three independent array analyses for each cell type before and after differentiation.

Data taken from Figure 4 were subjected to analyses identifying pathways, which might be targets of the proteins secreted by hsubMSC and hbmMSC before and after hepatocytic differentiation. Pathways affected comprised immune disease and cancer-related pathways as well as pathways involved in cellular processes. As expected, pathways involved were mainly identical for both hsubMSC and hbmMSC. Generally, the number of proteins engaged in the pathways increased after hepatocytic differentiation, corroborating that undifferentiated MSC were equipped with a basic profile of secretory proteins, tackling each single pathway, up-regulated by hepatocytic differentiation (Figure 5).

**Figure 6:**
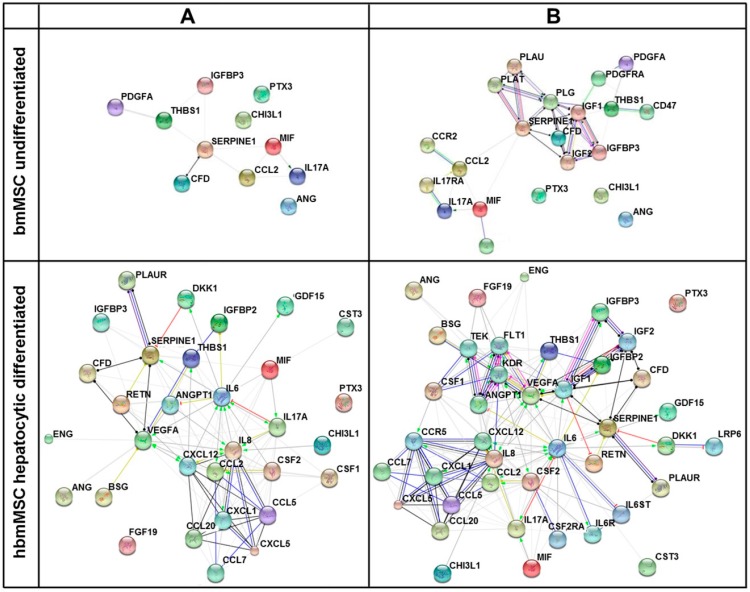
Network of interacting cytokines (**A**) and additional 10 predicted potential interaction partners (**B**) of undifferentiated (**upper** panels) and hepatocytic differentiated (**lower** panels) hbmMSC. Networks were created by the STRING database using only proteins secreted to high abundance as summarized in Tables S1 and S3. Connections between partners are shown in different colours; **green**: activation, **red**: inhibition, **blue**: binding, **cyan**: phenotype, **violet**: catalysis, **pink**: posttranslational modification, **black**: reaction, **yellow**: expression. Bubble coloursare only for a better discrimination.

**Figure 7:**
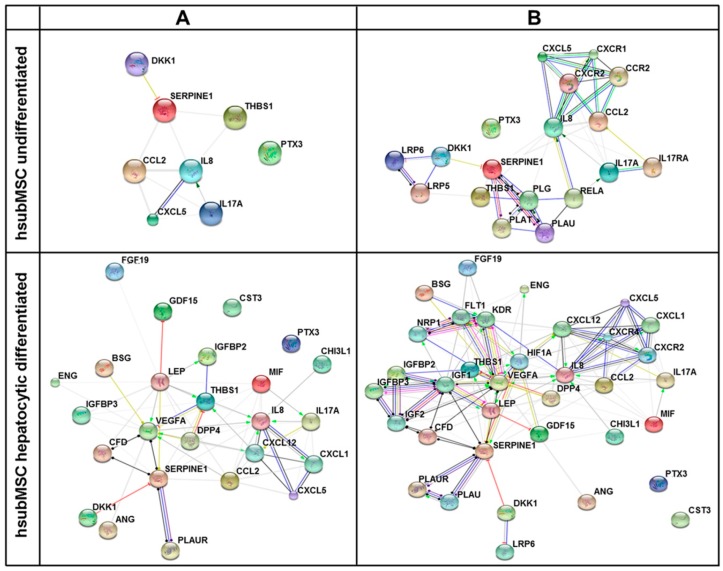
Network of interacting cytokines (**A**) and additional 10 predicted potential interaction partners (**B**) of undifferentiated (**upper** panels) and hepatocytic differentiated (**lower** panels) hsubMSC. Networks were created by the STRING database using only proteins secreted at high abundance as summarised in Tables S2 and S4. Connections between partners are shown in different colours; **green**: activation, **red**: inhibition, **blue**: binding, **cyan**: phenotype, **violet**: catalysis, **pink**: posttranslational modification, **black**: reaction, **yellow**: expression. Bubble colours are only for a better discrimination.

In order to achieve a higher resolution of putative biological networks, which might be significantly impacted by the factors found enriched in supernatants of hsubMSC and hbmMSC before and after their hepatocytic differentiation, the highly abundant proteins as summarised in Supplementary Materials Tables S1–S4 were subjected to networks analyses including the prediction of 10 potential interaction partners. The graphical summaries shown in Figure 6 and Figure 7 indicate that hepatocytic differentiation of both cell populations increased the number of potential interaction partners involved.

Even if at low or no significance, undifferentiated MSC might impact on pathways of both the acquired and the innate immunity like cytokine-cytokine receptor interactions (hsa04060, Kyoto Encyclopedia of Genes and Genomes (KEGG)) and the complement and coagulation cascade (hsa04610, KEGG) as well as the NOD-like receptor signalling pathway (Table 1).

These were highly significant after hepatocytic differentiation and new pathways emerged like the chemokine (hsa04062, KEGG) and the JAK-STAT signalling pathway as well as the Toll-like receptor pathway (map04620, KEGG), all involved in chemotactic and pattern recognition innate immune reactions. In addition, growth promoting and angiogenic pathways were highlighted like bladder cancer (hsa05219, KEGG), p53 signalling pathway (hsa04115, KEGG) and pathways in cancer (hsa05200, KEGG) (Table 2).

Taking into account prediction of 10 interaction partners, the identified pathways gained even higher significance thereby substantiating either their physiological relevance, or additional pathways, which were amended, featured growth and angiogenesis stimulation like glioma, melanoma or focal adhesion (Table 3 and Table 4).

Using the PathCards pathway unification database, the TGF-β Pathway Super Path was identified involving 686 genes in part regulated by factors secreted by hepatocytic differentiated hsubMSC and hbmMSC at low, medium and high abundance (PathCards pathway unification database), (Table 5). The TGF-beta pathway consists of a pathway network including the SOCS pathway, regulation of the eIF4 and p70S6K pathways, the TGF-β, JAK-STAT, JNK and MAPK pathways. 

## 3. Discussion

### 3.1. Paracrine Mechanisms in Liver Repair

MSC have been isolated from a wide variety of organisms and tissues and have been classified based on their multipotent differentiation capacity, ability to adhere to plastic substrata and expression of a specific set of mesenchymal surface markers [39,40]. Even if *bona fide* compatible with these criteria, it must be anticipated that cells from different sources may share similar but not identical functional features [41,42]. We corroborated this assumption by showing that MSC from human bone marrow and different sources of adipose tissue displayed the same surface marker profile, which changed after hepatocytic differentiation indicating a change in biological properties. According to their organ resident status, MSC are believed to support tissue regeneration by functional tissue replacement through differentiation into cells of the host organ/tissue or by secretion of paracrine factors, providing trophic support for self-regeneration of the tissue they reside in [7,43]. It is known that MSC secrete a multitude of soluble factors mediating pleiotropic actions such as immune-modulation, tissue regeneration or angiogenesis and others [26,27,44,45]. In summary, these features of MSC also seem to mediate the beneficial impact on both acute and chronic liver diseases. It was initially believed that the amelioration of liver damage by the stem cells required integration and functional specialization on the site of integration [46]. However, it became obvious, that paracrine factors, which have not been identified unequivocally so far, were the major principle of action [21,22,23,47]. Therefore, the trophic effect of MSC on the liver can probably be attributable to paracrine anti-inflammatory, immune-modulatory, anti-apoptotic and pro-proliferative actions augmenting self-regeneration of the liver [48,49].

Here, we compared secretion of factors from bone marrow- and adipose tissue-derived MSC before and after hepatocytic differentiation. MSC from different sources secreted a similar albeit not identical pattern of factors, corroborating previous results from bone marrow- and umbilical cord blood-derived MSC [50]. Pathway analyses of highly expressed factors revealed a strong correlation with processes engaged in liver regeneration such as cytokine/chemokine pathways regulating hepatic and extrahepatic immune reactions as well as inflammation, growth factor pathways regulating initiation and termination of hepatocyte proliferation, factors involved in the complement and coagulation cascade as well as in epithelial-to-mesenchymal transition (EMT) and angiogenesis, all required for morphogenic and parenchymal remodelling after chronic injury like fibrosis and cirrhosis. Finally, the association with the TGF-β pathway, which in the liver is involved in hepatocyte proliferation and differentiation after acute liver damage as well as in cell death and fibrotic tissue remodelling in the pathogenesis of chronic liver diseases [51], indicates that MSC-derived molecules may extensively interfere with both parenchymal and non-parenchymal tissue homeostasis in the liver.

### 3.2. Functional Relevance

IL-17A, MCP-1, Pentraxin 3, Serpin E1 and Thrompospondin-1 were mainly expressed by both undifferentiated bone marrow- and adipose tissue-derived MSC. IL-17A, a pro-inflammatory cytokine produced by Th17 and innate immune cells, protects the host from extracellular pathogens by the recruitment of immune cells like neutrophils. While poorly active on its own, IL-17 synergises with IL-1β, IL-22, IFNγ and GM-CSF supporting the host defence reaction by the augmentation of pro-inflammatory cytokines such as IL-6 and IL-8 [52]. A similar autocrine mechanism may underlie the increase in expression of these factors after hepatocytic differentiation of MSC as observed here.

Pentraxin 3 was expressed at high abundance under all conditions tested here (Figure 2). As a member of the long pentraxin family, it plays an essential part in the regulation of innate immunity, inflammation, complement activation and matrix deposition [53]. Also, Pentraxin 3 deficiency was associated with an enhanced inflammatory response and tissue damage [53], thus corroborating its essential role in tissue regeneration. As a key component of the innate immunity, Pentraxin 3 activated the downstream TLR4-MyD88 pathway during urinary tract infection [54]. The potential role of Pentraxin 3 in liver regeneration might be contributed to its interaction with FGF family members like FGF-2. Pentraxin 3 inhibited FGF-2-dependent endothelial cell proliferation and neovascularisation by the sequestration of FGF-2 [55]. The crosstalk with growth factor signaling, namely HGF and EGF, thus might link Pentraxin 3 functionally to the TGF-β pathway, which is the key player in liver morphogenesis and liver regeneration after partial hepatectomy, regulating both hepatocyte proliferation and growth termination [51,56]. Substantiating the impact of MSC on innate immune regulation, MCP-1 was mainly abundant in supernatants of undifferentiated MSC. In the injured liver, MCP-1 might originate from liver-resident macrophages, the Kupffer cells, to attract monocytes via the chemokine receptor CCR2. Normally involved in tissue remodelling and disease regression, inflammatory macrophages, however, might promote disease progression [57]. In line with its role in tissue remodelling as discussed above, soluble urokinase-type plasminogen activator receptor (uPAR) regulated the activity of MCP-1 and RANTES (CCL5) [58], which besides others regulate pattern recognition via NOD-like receptor signalling, thus coordinating innate immune activity with tissue homeostasis. The potential role of differentiated MSC in tissue remodelling is substantiated by the increase in CD54 (ICAM-1) expression (Figure 1). On human renal fibroblasts, ICAM-1 increased after activation by cross-linking the synthesis of RANTES and IL-8 [59], the latter acting as a chemo-attractant for granulocytes and is also abundant after differentiation of hbm- and hsubMSC as shown here. Moreover, on liver cells, ICAM-1 allows macrophages recruited by MCP-1 to adhere via the LFA-1 ligand [60]. This might also substantiate that the MSC differentiated into the hepatocytic lineage, which is also corroborated by the decrease of CD166 expressed as mesenchymal stem cell marker on liver fibroblasts [61].

Serpin E1, also known as plasminogen activator inhibitor-1 (PAI-1), is part of the fibrolytic system, and as such contributes to tissue remodelling after partial hepatectomy [62], angiogenesis and tumour progression [63]. It is a major acute phase reactant [64] and its expression is strongly enhanced by inflammatory stimuli [65]. In rat hepatocytes, the artificial glucocorticoid dexamethasone increased TGFβ-induced Serpin E1 expression [64] connecting Serpin E1 with the regulation of epithelial-to-mesenchymal transition (EMT) promoted by TGF-β [66]. Insulin and dexamethasone, two ingredients of the medium used in this study, are strong inducers of Serpin E1 expression. Insulin increased Serpin E1 expression via the MAPK or the phosphoinositide-3-kinase–protein kinase B (PI3K/PKB) pathway and indirectly via HIF1α [64]. Serpin E1 and uPAR, expressed by both bone marrow- and adipose tissue-derived MSC, are targets of HIF1α. While HIF1α promotes upregulation of growth factors like FGF-2 and HGF, HIF2α induces VEGFA, all of which are known to support wound healing [58]. In addition, Serpin E1 was reported to stimulate cell migration by the low density lipoprotein receptor-related protein 1 (LRP1)-dependent activation of the Wnt/β-catenin and ERK1/2-MAPK pathways [63]. In line with the impact of MSC on tissue remodelling after chronic liver disease, the inhibitor of Wnt-signalling, Dickkopf-1 (Dkk-1), highly abundant in supernatants of undifferentiated MSC, might foster resolution of fibrosis by the down-regulation of hepatic stellate cell activation [67]. Thus, Serpin E1 secreted by MSC seems to contribute to tissue remodelling and morphogenesis, thereby promoting liver regeneration after injury. In contrast, Thrombospondin-1, highly expressed by undifferentiated hsub- and hbmMSC, has been shown to suppress VEGF activity and hepatocyte growth through TGF-β-dependent mechanisms [31,68], thus antagonising liver regeneration. Indeed, platelet-derived α-granules contained both Thrombospondin-1 and VEGF, and human data demonstrated that high Thrombospondin-1 and low VEGF were predictors of liver dysfunction after resection [69].

Hepatocytic differentiation of both hsubMSC and hbmMSC further increased secretion of the factors as discussed above and moreover a wide variety of proteins, which might interfere with different pathways involved in the maintenance of liver architectural and functional homeostasis. To discuss a selection, VEGF as well as angiopoietins 1 and 2 are important promoters of liver regeneration [30]. Platelet-derived growth factor AA (PDGF-AA), albeit expressed in low abundance, is mainly produced by plateletsin vivo. Its hepatotropic properties have been corroborated by the transplantation of platelets improving liver regeneration after resection in rats [70] and in patients after living donor transplantation [71]. FGF-19 might stimulate insulin-dependent pathways regulating hepatic protein and glycogen metabolism [72]. The neurotrophin BDNF mediated cell survival and repair in the brain after ischemia [73]. It might act similarly in the liver since it has been shown that rat and human hepatic stellate cells and hepatocytes expressed BDNF and other neurotrophins involved in the pathogenesis of liver diseases [74]. The expression of chitinase 3-like 1 by hepatic stellate cells, which was positively associated with cell survival and negatively with liver fibrosis [75], might be enhanced by MSC-derived IL-6. Complement factor D, the rate limiting step of the alternative pathway of complement activation, may act as an adipokine, thus linking tissue homeostasis and metabolic regulation in chronic liver diseases like non-alcoholic steatohepatitis (NASH) [76,77]. Sex hormone-binding globulin (SHBG), mainly secreted by hepatocytic differentiated hsubMSC, is a liver-derived plasma protein, whose low levels were associated with non-alcoholic fatty liver disease (NAFLD) [78] and insulin resistance [79]. Stromal cell-derived factor 1 (SDF-1), stimulating homing to and differentiation of MSC at the site of injury [80] via its receptor C-X-C chemokine receptor type 4 (CXCR4), was highly expressed after hepatocytic differentiation, which might be due to the autocrine activation by Macrophage migration inhibitory factor (MIF) [81], also expressed in high levels after differentiation.

## 4. Experimental Section

### 4.1. Human Material

Human bone marrow was obtained during elective knee or hip joint surgery, human adipose tissue (subcutaneous, visceral and mesenteric) during abdominal surgery after receiving the patients´ written consent as approved by the Institutional Ethics Review Board Leipzig (file No. 282-11-22082011 and 282-10-04102010).

### 4.2. Mesenchymal Stem Cell Isolation, Propagation and Hepatocytic Differentiation

Human bone marrow and adipose tissue were collected in high glucose Dulbecco’s Modified Eagle’s medium (DMEM) (Gibco, Paisley, UK). Adipose tissue was further cut into pieces and tissues of both origins were subsequently incubated for 25 min at 37 °C with collagenase (0.2 unit/mL, NB4G, Serva, Heidelberg, Germany). Digestion was stopped by addition of 5 mL fetal bovine serum (Gibco, Paisley, UK) followed by several washing steps in phosphate-buffered saline (PBS). MSC were enriched by density gradient centrifugation, propagated and cryopreserved essentially as described. Upon thawing, cells were seeded (400 cells/cm^2^) onto human fibronectin-coated culture dishes in growth medium and cultured until reaching a confluence of 80%–90% (7–10 days). These cells were used for analyses of undifferentiated MSC. Hepatocytic differentiation was initiated by continuing culture with 5′-Azacytidine for another 24 h. Thereafter, the medium was changed and culture proceeded in human hepatocyte medium supplemented with 2% fetal calf serum, HGF and EGF routinely until day 16 of hepatocytic differentiation as described in detail in [19]. Analyses were performed with non-pooled and non-passaged stocks of MSC from the number of donors as given in the legends to the figures.

### 4.3. Microscopic Documentation of Morphology

Morphology of the undifferentiated MSC from different origins was documented using the phase contrast microscope Primo Vert with the Zen software (Zeiss, Jena, Germany).

### 4.4. Flow Cytometry

After correction for the IgG isotype control, surface marker profiles of undifferentiated and hepatocytic differentiated human bone marrow- and adipose tissue-derived MSC were captured by flow cytometry using the LSR II FACS Diva 8.0.1 software (Becton Dickinson Bioscience, San Jose, CA, USA). Data were analysed by Kathrin Jäger and Andreas Lösche at the Core Unit Fluorescence Technologies, Interdisciplinary Centre for Clinical Research (IZKF), University Leipzig. Experimental details are described elsewhere [18,19,82] and antibodies used are listed in Table S6. Dead cells were excluded from the measurements by propidium iodide staining. For the sake of comparability with our previous studies, differentiation was continued until day 21 of hepatocytic differentiation [18,19]. Data shown are means of 3–5 analyses with cells from different donors.

### 4.5. Sample Preparation and Proteome Profiler Antibody Array

Proteome analyses were performed with bone marrow- and subcutaneous adipose tissue-derived MSC from three different donors each. Undifferentiated cells were cultured in growth medium supplemented with 15% inactivated fetal bovine serum until 65% confluence. After washing with PBS for three times, culture was continued in growth medium without serum addition. After another four days without medium change, supernatants were collected for analyses (cell numbers—hbmMSC: 350,000 ± 24,000, hsubMSC: 450,000 ± 80,500, *p* = 0.22). Hepatocytic differentiated MSC were grown for 12 days in human hepatocyte medium as described above. Then the cells were washed three times with PBS and culture continued for another 4 days in serum-free human hepatocyte medium before the supernatants were collected for analyses (cell numbers—hbmMSC: 470,000 ± 40,000, hsubMSC: 880,000 ± 78,000, *p* = 0.0002). To assess background reactions, negative controls without cells were run under both culture settings.

Supernatants were centrifuged for 5 min (4 °C, 1.5× *g*) and 300 µL of each sample were incubated overnight with binding buffer according to the manufacturer’s instructions for use of the Proteome Profiler™ Human XL Cytokine Array Kit (R&D Systems, Minneapolis, MN, USA), which detects 102 proteins as listed in Supporting Information Table 2. After washing, membranes were incubated with the detection cocktail for 1 h, followed by washing steps, incubation with streptavidin-conjugated horseradish peroxidase (HRP, R&D Systems, Minnesota, MN, USA) for 30 min, another washing steps, and finally the incubation with the chemiluminescence reagent for 1 min. Abbreviations of proteins as analysed by the arrays are listed under Table S6.

### 4.6. Array Analysis and Bioinformatics

For bioinformatics analyses, labelled proteins were visualized by the Micro Chemi 4.2 using the gel capture software (Biostep, Burkhardtsdorf, Germany). Serial pictures with an exposure time of 9 min were quantified using Image J 1.46 (NIH, Bethesda, MD, USA) and corrected for background signals of the negative controls. Mean pixel density of reference spots was set to 100, to which all other values given are relative. During hepatocytic differentiation cell number increased as described above due to the presence of HGF and EGF. Therefore, results were corrected for the number of hbmMSC and hsubMSC before and after hepatocytic differentiation. Accounting for the fact that the medium contained some ingredient proteins cross-reacting with the array, proteins with a relative mean pixel density below 5 were categorized as “not abundant” (unspecific background signals), between 5 and 15 as “low abundant”, between 15 and 30 as “medium abundant” and above 30 as “high abundant”. Predicted interactions of the cytokine network of high abundance analytes (entered in the ENTREZ GENE ID format) as well as the association with another 10 predicted potential interaction partners were visualised using the online STRING database platform (http://string-db.org/) [83], which was also used for predicted pathway analysis of all abundant analytes. Predicted pathway analysis of high abundant analytes was performed by the DAVID Bioinformatics Resources 6.7 database (https://david.ncifcrf.gov/) [84,85] and associated with KEGG-pathways. The TGF-β Pathway Super Path and corresponding genes were assessed by using the PathCards pathway unification database (http://pathcards.genecards.org/) [86].

### 4.7. Statistics

Experiments were repeated at least three times. Results are shown as means ± standard error of the mean. If not otherwise mentioned, Student’s *t*-test was used to confirm significant differences at the level of *p* as indicated in the legends to the figures.

## 5. Conclusions

The compilation of factors produced by human MSC from different tissue sources demonstrated that the manipulation of the cells like hepatocytic differentiation dramatically changed the pattern of the secreted proteins, both qualitatively and quantitatively. The comprehensive analysis of factors and their targeted pathways unravelled a variety of hepatotropic networks involved in the regulation of tissue and functional homeostasis during the pathogenesis and regression of liver diseases. Pathways addressed by both undifferentiated MSC and hepatocytic differentiated MSC comprised the innate and adaptive immunity, proliferation and apoptosis, liver regeneration, the complement and coagulation pathway as well as cytokine and chemokine pathways. It is evident from our study that these pathways and the respective factors secreted by human MSC regulating these pathways are tentatively involved in hepatic repair after injury, but may also contribute to disease progression. It is hence of utmost importance before clinical applications, to determine the pattern of factors secreted by a specific MSC population, both native and after manipulation, in order to delineate and predict the potential impact on liver diseases. It is also evident that the pattern of factors secreted by MSC may be impacted by the specific liver disease, which is targeted by MSC therapy. It must be anticipated, that host- and donor MSC-derived paracrine and autocrine loops imprint the secretory pattern of MSC, which may result in either disease aggravation or amelioration [87]. Even if possible, the breakdown of MSC action to single molecular pathways to be addressed as therapeutic targets seems neither reasonable nor reliable for the use of MSC in treating liver diseases, since the pleiotropic actions of MSC rely on the intersection with disease-tailored signals and networks generated by the diseased host liver. Knowledge of these intersections as provided by our study will facilitate the individualised application and the prediction of the most assumable success of MSC therapy for liver diseases.

## Figures and Tables

**Table 1:** Pathway analysis by the David database of high abundance analytes secreted by undifferentiated hbmMSC and hsubMSC (taken from Tables S1 and S2). Associated KEGG-pathways with *p*-values of significance as well as analytes involved and “not found” are shown, respectively.

hbmMSC, Undifferentiated—High Abundance Analytes			
Associated KEGG-Pathway	*p*-Value	Analytes Involved (Entrez Gene ID)	Analytes Not Found (Entrez Gene ID)
p53 signalling pathway	3.5 × 10^−3^	IGFBP-3 (3486) Thromposondin-1 (7057) Serpin E1 (5054)	Angiogenin (283) Chitinase 3-like 1 (1116) MIF (4282) Pentraxin-3 (5806)
Cytokine-cytokine receptor interaction	4.7 × 10^−2^	MCP-1 (6347) IL-17A (3605) PDGF-AA (5154)	
Complement and coagulation cascade	9.1 × 10^−2^	Serpin E1 (5054) Complement factor D (1675)	
**hsubMSC, Undifferentiated—High Abundance Analytes**			
**Associated KEGG-Pathway**	***p*-Value**	**Analytes Involved (Entrez Gene ID)**	**Analytes Not Found (Entrez Gene ID)**
Cytokine-cytokine receptor interaction	2.4 × 10−3	MCP-1 (6347) ENA-78 (6374) IL-17A (3605) IL-8 (3576)	Dkk-1 (22,943) Pentraxin-3 (5806)
Chemokine signalling pathway	1.8 × 10−2	MCP-1 (6347) ENA-78 (6374) IL-8 (3576)	
Bladder cancer	4.9 × 10−2	IL-8 (3576) Thromposondin-1 (7057)	
NOD-like receptor signalling pathway	7.1 × 10−2	IL-8 (3576) MCP-1 (6347)	
p53 signalling pathway	7.8 × 10−2	Thromposondin-1 (7057) Serpin E1 (5054)	

**Table 2:** Pathway analysis by the David database of high abundance analytes secreted by hepatocytic differentiated hbmMSC and hsubMSC (taken from Tables S3 and S4). Associated KEGG-pathways with *p*-values of significance as well as analytes involved and “not found” are shown, respectively.

hbmMSC, Hepatocytic Differentiated—High Abundance Analytes			
Associated KEGG-Pathway	*p*-Value	Analytes Involved (Entrez Gene ID)	Analytes Not Found (Entrez Gene ID)
Cytokine-cytokine receptor interaction	2.4 × 10^−11^	MCP-1 (6347) MCP-3 (3654) MIP-3α (6364) RANTES (6352) GRO-α (2919) SDF-1 α (6387) ENA-78 (6374) M-CSF (1435) GM-CSF (1437) IL-17A (3605) IL-6 (3569) IL-8 (3576) VEGF (7422)	Angiogenin (283) Angiopoetin-1 (284) EMMPRIN (682) Chitinase 3-like 1 (1116) Cystatin C (1471) Dkk-1 (22,943) Endoglin (2022) FGF-19 (9965) GDF-15 (9518) IGFBP-2 (3485) MIF (4282) Pentraxin-3 (5806) Resistin (56,729)
NOD-like receptor signalling pathway	3.1 × 10^−6^	MCP-1 (6347) RANTES (6352) MCP-3 (6354) GRO-α (2919) IL-6 (3569) IL-8 (3576)	
Chemokine signalling pathway	4.2 × 10^−6^	MCP-1 (6347) MIP-3α (6364) RANTES (6352) MCP-3 (6354) GRO-α (2919) SDF-1 α (6387) ENA-78 (6374) IL-8 (3576)	
Bladder cancer	1.2 × 10^−2^	IL-8 (3576) Thromposondin-1 (7057) VEGF (7422)	
p53 signalling pathway	2.9 × 10^−2^	IGFBP-3 (3486) Serpin E1 (5054) Trombospondin 1 (7057)	
Epithelial cell signalling in Heliobacter pylori infection	2.9 × 10^−2^	RANTES (6352) GRO-α (2919) IL-8 (3576)	
Complement and coagulation cascade	2.9 × 10^−2^	uPAR (5329) Serpin E1 (5054) Complement factor D (1675)	
Hematopoietic cell linage	4.4 × 10^−2^	M-CSF (1435) GM-CSF (1437) IL-6 (3569)	
Toll-like receptor signalling pathway	5.9 × 10^−2^	RANTES (6352) IL-6 (3569) IL-8 (3576)	
**hsubMSC, Hepatocytic Differentiated—High Abundance Analytes**			
**Associated KEGG-Pathway**	***p*-Value**	**Analytes Involved (Entrez Gene ID)**	**Analytes Not Found (Entrez Gene ID)**
Cytokine-cytokine receptor interaction	4.0 × 10^−6^	MCP-1 (6347) GRO-α (2919) SDF-1 α (6387) ENA-78 (6374) IL-17A (3605) IL-8 (3576) Leptin (3952) VEGF (7422)	Angiogenin (283) EMMPRIN (682) Chitinase 3-like 1 (1116) Cystatin C (1471) Dkk-1 (22,943) DPPIV (1803) Endoglin (2022) GDF-15 (9518) IGFBP-2 (3485) MIF (4282) Pentraxin-3 (2806) FGF-19 (9956)
Chemokine signalling pathway	1.8 × 10^−3^	MCP-1 (6347) GRO-α (2919) SDF-1 α (6387) ENA-78 (6374) IL-8 (3576)	
Bladder cancer	6.5 × 10^−2^	IL-8 (3576) Thromposondin-1 (7057) VEGF (7422)	
NOD-like receptor signalling pathway	1.4 × 10^−2^	MCP-1 (6347) GRO-α (2919) IL-8 (3576)	
p53 signalling pathway	1.7 × 10^−2^	IGFBP-3 (3486) Thromposondin-1 (7057) Serpin E1 (5054)	
Complement and coagulation cascade	1.7 × 10^−2^	uPAR (5329) Serpin E1 (5054) Complement factor D (1675)	

**Table 3:** Pathway analysis by the David database of high abundance analytes secreted by undifferentiated hbmMSC and hsubMSC and 10 potential interaction partners as predicted by the STRING database (input of analytes as summarised in Tables S1 and S2). Associated KEGG-pathways with *p*-values of significance as well as analytes involved are shown.

hbmMSC, Undifferentiated—High Abundance Analytes			
Associated KEGG-Pathway	*p*-Value	Analytes Involved (Entrez Gene ID)	Predicted Genes (Entrez Gene ID)
Complement and coagulation cascades	3.8 × 10^−5^	SerpinE1 (5054) Complement factor D (1675) PLAT (5327) PLG (5340) PLAU (5328)	IGF1 (3479) IGF2 (3481) PLAU (5328) IL-17RA (23,765) CCR2 (1231) PLAT (5327) PLG (5340) PRDM1 (639) PDGFRA (5156) CD47 (961)
p53 signalling pathway	9.3 × 10^−4^	IGF1 (3479) IGFBP-3 (3486) Serpin E1 (5054) Thrombospondin 1 (7057)	
Prostate cancer	2.0 × 10^−3^	IGF1 (3479) IGF2 (3481) PDGF AA (5154) PDGFRA (5156)	
Cytokine-cytokine receptor interaction	6.0 × 10^−3^	PDGF AA (5154) PDGFRA (5156) IL-17 (3506) IL-17RA (23,765) MCP-1 (6347)	
Glioma	1.4 × 10^−2^	IGF1 (3479) PDGF AA (5154) PDGFRA (5156)	
Melanoma	1.8 × 10^−2^	IGF1 (3479) PDGF AA (5154) PDGFRA (5156)	
Focal adhesion	1.9 × 10^−2^	IGF1 (3479) PDGF AA (5154) PDGFRA (5156) Thrombospondin 1 (7057)	
**hsubMSC, Undifferentiated-High Abundance Analytes**			
**Associated KEGG-Pathway**	***p*-Value**	**Analytes Involved (Entrez Gene ID)**	**Predicted Genes (Entrez Gene ID)**
Cytokine-cytokine receptor interaction	6.0 × 10^−5^	IL-17 (3506) IL-17RA (23,765) MCP-1 (6347) ENA-78 (6374) IL-8 (3576) CXCR1 (3577) CXCR2 (3579)	LRP5 (4041) LRP6 (4040) CXCR1 (3577) CXCR2 (3579) PLAU (5328) PLAT (5327) PLG (5340) CCR2 (1231) IL-17RA (23,765) RELA (5970)
Chemokine signalling pathway	1.4 × 10^−4^	MCP-1 (6347) ENA-78 (6374) IL-8 (3576) CXCR1 (3577) CXCR2 (3579) RELA (5970)	
Epithelial cell signalling in Helicobacter pylori infection	9.3 × 10^−4^	IL-8 (3576) CXCR1 (3577) CXCR2 (3579) RELA (5970)	
Complement and coagulation cascades	9.7 × 10^−4^	PLAU (5328) PLAT (5327) PLG (5340) Serpin E1 (5054)	
NOD-like receptor signalling pathway	1.4 × 10^−2^	MCP-1 (6347) RELA (5970) IL-8 (3576)	
Wnt signalling pathway	7.1 × 10^−2^	Dkk-1 (22,943) LRP5 (4041) LRP6 (4040)	

**Table 4:** Pathway analysis by the David database of high abundance analytes secreted by hepatocytic differentiated hbmMSC and hsubMSC and 10 potential interaction partners as predicted by the STRING database (input of analytes as summarised in Tables S3 and S4). Associated KEGG-pathways with *p*-values of significance as well as analytes involved are shown.

hbmMSC, Hepatocytic Differentiated—High Abundance Analytes			
Associated KEGG-Pathway	*p*-Value	Involved Analytes (Entrez Gene ID)	Predicted Genes (Entrez Gene ID)
Cytokine-cytokine receptor interaction	1.8 × 10^−16^	MCP-1 (6347) MIP-3 α (6364) RANTES (6352) MCP-3 (6354) CCR5 (1234) Gro-α (2919) SDF-1 α (6387) ENA-78 (6374) M-CSF (1435) GM-CSF (1437) CSF2RA (1438) FLT1 (2321) IL-17 (3506) IL-6 (3569) IL-6R (3570) IL-6ST (3572) IL-8 (3576) KDR (3791) VEGF A (7422)	KDR (3791) FLT1 (2321) IGF1 (3479) IL-6R (3570) LRP6 (4040) CCR5 (1234) CSF2RA (1438) IL-6ST (3572) NRP1 (8829) IGF2 (3481)
Chemokine signalling pathway	8.4 × 10^−6^	MCP-1 (6347) MIP-3 α (6364) RANTES (6352) MCP-3 (6354) CCR5 (1234) Gro-α (2919) SDF-1 α (6387) ENA-78 (6374) IL-8 (3576)	
NOD-like receptor signalling pathway	2.6 × 10^−5^	MCP-1 (6347) RANTES (6352) MCP-3 (6354) Gro-α (2919) IL-6 (3569) IL-8 (3576)	
Hematopoietic cell lineage	1.5 × 10^−3^	M-CSF (1435) GM-CSF (1437) CSF2RA (1438) IL-6 (3569) IL-6R (3570)	
p53 signalling pathway	7.2 × 10^−3^	IGF1 (3479) IFGBP3 (3486) Serpin E1 (5054) Trombospondin 1 (7057)	
JAK-STAT signalling pathway	9.6 × 10^−3^	GM-CSF (1437) CSF2RA (1438) IL-6 (3569) IL-6R (3570) IL-6ST (3572)	
Bladder cancer	2.5 × 10^−2^	IL-8 (3576) Thrombospondin-1 (7057) VEGF A (7422)	
Focal adhesion	9.7 × 10^−2^	KDR (3791) FLT1 (2321) Thrombospondin 1 (7057) VEGF A (7422)	
mTOR signalling pathway	3.7 × 10^−2^	IGF1 (3479) IGF2 (3481) VEGF A (7422)	
Pathways in cancer	4.1 × 10^−2^	CFS2RA (1438) FGF-19 (9965) IGF1 (3479) IL-6 (3569) IL-8 (3576) VEGF A (7422)	
Epithelial cell signalling in Helicobacter pylori infection	6.0 × 10^−2^	RANTES (6352) Gro-α (2919) IL-8 (3576)	
Complement and coagulation cascades	6.2 × 10^−2^	uPAR (5329) Complement factor D (1675) Serpin E1 (5054)	
**hsubMSC, Hepatocytic Differentiated—High Abundance Analytes**			
**Associated KEGG-Pathway**	***p*-Value**	**Analytes Involved (Entrez Gene ID)**	**Predicted Genes (Entrez Gene ID)**
Cytokine-cytokine receptor interaction	1.3 × 10^−8^	KDR (3791) FLT1 (2321) MCP-1 (6347) Gro-α (2919) SDF-1 α (6387) ENA-78 (6374) IL-8 (3576) IL-17A (3605) CXCR2 (3579) CXCR4 (7852) Leptin (3952) VEGF A (7422)	KDR (3791) FLT1 (2321) IGF-1 (3479) LRP6 (4040) IGF-2 (3481) NRP1 (8829) PLAU (5328) CXCR4 (7852) HIF1A (3091) CXCR2 (3579)
Chemokine signalling pathway	2.3 × 10^−4^	MCP-1 (6347) MIP-3 α (6364) Gro-α (2919) SDF-1 α (6387) ENA-78 (6374) IL-8 (3576) CXCR2 (3579) CXCR4 (7852)	
mTOR signalling pathway	2.0 × 10^−3^	HIF1A (3091) IGF1 (3479) IGF2 (3481) VEGF A (7422)	
p53 signalling pathway	4.3 × 10^−3^	IGF-1 (3479) IGFBP-3 (3486) Serpin E1 (5054) Thrombospondin 1 (7057)	
Complement and coagulation cascades	4.4 × 10^−3^	uPAR (5329) PLAU (5328) Complement factor D (1675) Serpin E1 (5054)	
Focal adhesion	1.6 × 10^−2^	KDR (3791) FLT1 (2321) IGF-1 (3479) Thrombospondin 1 (7057) VEGF A (7422)	
Bladder cancer	1.8 × 10^−2^	IL-8 (3576) Thrombospondin 1 (7057) VEGF A (7422)	
NOD-like receptor signalling pathway	3.7 × 10^−2^	MCP-1 (6347) Gro-α (2919) IL-8 (3576)	
Epithel cell signalling in Heliobacter pylori infection	4.3 × 10^−2^	Gro-α (2919) IL-8 (3476) CXCR2 (3579)	
Endocytosis	6.0 × 10^−2^	CXCR4 (7852) IL-8 (3576) FLT1 (2321) KDR (3791)	
Pathways in cancer	7.4 × 10^−2^	IGF-1 (3479) HIF1A (3091) FGFR2 (2263) FGF-7 (2252) FGF-19 (9965) IL-6 (3569) IL-8 (3576) VEGF A (7422)	

**Table 5:** Analytes secreted by hepatocytic differentiated hbm- and hsub-MSC in association with the TGF-β Pathway Super Path as identified by the PathCards pathway unification database.

TGF-β Pathway-Related Analytes			
Cell Type	Low Abundance	Medium Abundance	High Abundance
**hbmMSC, hepatocytic differentiated**	BAFF BDNF IL-1α IL-1β IL-2 IL-3 IL-10 IL-13 IL15 IL-16 IL-19 IL-24 MIP-1α/MIP-1β MIP-3β PDGF-AA TARC TGFα	Angiopoetin-2 FGF basic FGF-7 IL-4 IL-11 IL-22	Angiopoetin-1 FGF-19 GDF-15 IL-17 IL-6 IL-8 MCP-1 MCP-3 MIP-3α RANTES SDF-1α
**hsubMSC, hepatocytic differentiated**	Angiopoetin-1 Angiopoetin-2 BAFF FGF basic IL-1α IL-1β IL-10 IL-11 IL-24 MCP-3 PDGF-AA RANTES TARC	BDNF FGF-7 IL-4 IL-6 IL-22 MIP-3α	FGF-19 GDF-15 IL-8 IL-17A MCP-1 SDF-1α

## Supplementary Materials

Supplementary materials can be found at http://www.mdpi.com/1422-0067/17/7/1099/s1.

## Abbreviations

hbmMSC	Human bone marrow-derived mesenchymal stem cells
hsubMSC	Human subcutaneous adipose tissue-derived mesenchymal stem cells
