# Supplementary material for: Identification of Pathways in Liver Repair Potentially Targeted by Secretory Proteins from Human Mesenchymal Stem Cells
Source: Int J Mol Sci. 2016 Jul 9;17(7):1099. doi: 10.3390/ijms17071099 (PMC4964475; doi:10.3390/ijms17071099)
Supplement: Supplementary file 1 [file ijms-17-01099-s001.pdf]

Supplementary Materials: Identification of Pathways in Liver Repair Potentially Targeted by Secretory Proteins from Human Mesenchymal Stem Cells

Sandra Winkler, Madlen Hempel, Sandra Brückner, Hans-Michael Tautenhahn, Roland Kaufmann and Bruno Christ

Table S1. Arbitrary abundance of proteins secreted by undifferentiated human bone marrow-derived mesenchymal stem cells (hbmMSC). Italic script indicates overlapping proteins in the same category (low, medium or high abundance) as in human subcutaneous adipose tissue-derived stem cells (cf. Table S2).

**hbmMSC, Undifferentiated Protein Abundance**		
**+ Low**	**++ Medium**	**+++ High**
Angiopoetin-2		
CD14		
FGF basic		
FGF-7	Angiopoetin-1	Angiogenin
HGF	Cystatin C	Chitinase 3-like 1
IL-2	Dkk-1	Complement factor D
IL-4	EMMPRIN	IGFBP-3
IL-6	ENA-78	*IL-17A*
IL-10	Endoglin	*MCP-1*
IL-11	FGF-19	MIF
IP-10	GDF-15	PDGF-AA
Leptin	IGFBP-2	*Pentraxin-3*
M-CSF	IL-22	*Serpin E1*
RAGE	μPAR	*Thrombospondin-1*
RANTES		
SDF-1α		
VEGF		
Vitamin D BP		

Table S2. Arbitrary abundance of proteins secreted by undifferentiated human subcutaneous adipose tissue-derived mesenchymal stem cells (hsubMSC). Italic script indicates overlapping proteins in the same category (low, medium or high abundance) as in human bone marrow-derived stem cells (cf. Table S1).

**hsubMSC, Undifferentiated Protein Abundance**		
**+ Low**	**++ Medium**	**+++ High**
Angiogenin		
Complement factor D		
CRP		
EGF		
FGF basic		
FGF-19		
GM-CSF		
Gro-α		Dkk-1
ICAM-1		ENA-78
IGFBP-3		IL-8
IL-1β	IL-6	*IL-17A*
IL-2		*MCP-1*
IL-11		*Pentraxin-3*
IL-12p70		*Serpin E1*
MIF		*Thrombospondin-1*
Osteopontin		
PDGF-AA		
RANTES		
RBP4		
Resistin		
μPAR		
Vitamin D BP		

Table S3. Arbitrary abundance of proteins secreted by hbmMSC after 16 days of hepatocytic differentiation. Italic script indicates overlapping proteins in the same category (low, medium or high abundance) as in human subcutaneous adipose tissue-derived stem cells (cf. Table S4).

**hbmMSC, Hepatocytic Differentiated Protein Abundance**		
**+ Low**	**++ Medium**	**+++ High**
Aggrecan		*Angiogenin*
BAFF		Angiopoetin-1
BDNF		*Chitinase 3-like 1*
Complement component C5/C5s	Angiopoetin-2	*Complement factor D*
CD30	CD14	*Cystatin C*
CD40Ligand	DPPIV	*Dkk-1*
CRP	FGF basic	*EMMPRIN*
Cripto-1	*FGF-7*	*ENA-78*
Fas Ligand	*IL-4*	*Endoglin*
Flt-3 Ligand	IL-11	*FGF-19*
G-CSF	*IL-22*	*GDF-15*
ICAM-1	IP-10	GM-CSF
IFN-γ	Vitamin D BP	*Gro-α*
IL-1α		*IGFBP-2*
IL-1β		*IGFBP-3*
IL-1α receptor		IL-6
		IL-8

IL-2	*IL-17A*
IL-3	*MCP-1*
IL-10	MCP-3
IL-12p70	M-CSF
IL-13	*MIF*
IL-15	MIP-3α
IL-16	*Pentraxin-3*
IL-18BPa	RANTES
IL-19	Resistin
IL-23	*SDF-1α*
IL-24	*Serpin E1*
IL-27	*Thrombospondin-1*
IL-31	*uPAR*
IL-32α/β/γ	*VEGF*
IL-33	
IL-34	
I-TAC	
Kallikrein-3	
Leptin	
LIF	
Lipocalin-2	
MIG	
MIP-1α/MIP-1β	
MIP-3β	
MMP-9	
Osteopontin	
PDGF-AA	
PF4	
RAGE	
RBP4	
Relaxin-2	
SHBG	
ST2	
TARC	
TFF3	
TfR	
TGF-α	
TNF-α	

Table S4. Arbitrary abundance of proteins secreted by hsubMSC after 16 days of hepatocytic differentiation. Italic script indicates overlapping proteins in the same category (low, medium or high abundance) as in human bone marrow-derived stem cells (cf. Table S3).

**hsubMSC, Hepatocytic Differentiated Protein Abundance**		
**+ Low**	**++ Medium**	**+++ High**
Angiopoetin-1		
Angiopoetin-2		
BAFF		
Complement component		
C5/C5a		
CD14		
CD40 Ligand		
CRP		*Angiogenin*
FGF basic		*Chitinase 3-like 1*
Flt-3 Ligand		*Complement factor D*
G-CSF		*Cystatin C*
GM-CSF		*Dkk-1*
IFN-γ		DPPIV
IL-1α		*EMMPRIN*
IL-1β		*ENA-78*
IL-1α receptor	BDNF	*Endoglin*
IL-10	*FGF-7*	*FGF-19*
IL-11	*IL-4*	*GDF-15*
IL-24	IL-6	*Gro-α*
IL-27	*IL-22*	*IGFBP-2*
IL-32	MIP-3α	*IGFBP-3*
α/β/γ	SHBG	*IL-8*
IP-10	ST2	*IL-17A*
Kallikrein-3		Leptin
MCP-3		*MCP-1*
M-CSF		*MIF*
MIG		*Pentraxin-3*
MMP-9		*SDF-1α*
PDGF-AA		*Serpin E1*
PF4		*Thrombospondin-1*
RAGE		*uPAR*
RANTES		*VEGF*
RBP4		
Relaxin-2		
Resistin		
TARC		
TFF3		
TfR		
Vitamin D BP		

Table S5. Analytes, alternative names and Entrez Gene IDs of the Human XL Cytokine Array as listed in the appendix of the manufacturer´s manual. The last column shows the numbers as applied for designation of radii in Figure 4.

Protein	Alternative Names	Entrez Gene ID	Secretome Profile ID (Figure 4)
Adiponectin	Acrp30	9370	1
Aggrecan	Aggrecan1	176	2
Angiogenin	---	283	3
Angiopoietin-1	Ang-1, ANGPT1	284	4
Angiopoietin-2	Ang-2, ANGPT2	285	5
BAFF	Blys, TNFSF13B	10,673	6
BDNF	Brain-derived Neurotrophic Factor	627	7
Complement Component C5/C5a	C5/C5a	727	8
CD 14	---	929	9
CD 30	TNFRSF8	943	10
CD 40 ligand	CD40L, TNFSF5, CD154, TRAP	959	11
Chitinase 3-like 1	CHI3L1, YKL-40	1116	12
Complement Factor D	Adipsin, CFD	1675	13
CRP	C-Reactive Protein	1401	14
Cripto-1	Teratocarcinoma-derived Growth Factor	6997	15
Cystatin C	CST3, ARMD11	1471	16
Dkk-1	Dickkopf-1	22,943	17
DPPIV	CD26, DPP4, Dipeptidy-pedidase IV	1803	18
EGF	Epidermal Growth Factor	1950	19
EMMPRIN	CD147, Basigin	682	20
ENA-78	CXCL5	6374	21
Endoglin	CD105, ENG	2022	22
Fas Ligand	TNFSF6, CD178, CD95L	356	23
FGF basic	FGF-2	2247	24
FGF-7	KGF	2252	25
FGF-19	---	9965	26
Flt-3 Ligand	FLT3LG	2323	27
G-CSF	CSF3	1440	28
GDF-15	MIC-1	9518	29
GM-CSF	CSF2	1437	30
GRO-α	CXCL1, MSGA-α	2919	31
Growth Hormone	GH, Somatotropin	2688	32
HGF	Scatter Factor, SF	3082	33
ICAM-1	CD54	3383	34
IFN-γ	IFNG	3458	35
IGFBP-2	---	3485	36
IGFBP-3	---	3486	37
IL-1α	IL-1F1	3552	38
IL-1β	IL-1F2	3553	39
IL-1ra	IL-1F3	3557	40
IL-2	---	3558	41
IL-3	---	3562	42
IL-4	---	3565	43
IL-5	---	3567	44
IL-6	---	3569	45
IL-8	CXCL8	3576	46
IL-10	---	3586	47
IL-11	---	3589	48
IL-12p70	---	3593	49
IL-13	---	3596	50
IL-15	---	3600	51
IL-16	---	3603	52
IL-17A	IL-17, CTLA8	3605	53
IL-18 BPa	---	10,068	54
IL-19	---	29,949	55
IL-22	IL-TIF	50,616	56
IL-23	IL-23A, SGRF	51,561	57
IL-24	C49A, FISP, MDA-7, MOB-5, ST16	3627	58

Table S5. *Cont.*

Protein	Alternative Names	Entrez Gene ID	Secretome Profile ID (Figure 4)
IL-27	---	246,778	59
IL-31	---	386,653	60
IL-32α/β/γ	---	9235	61
IL-33	C9orf26, DVS27, NF-HEV	90,865	62
IL-34	C9orf77	146,433	63
IP-10	CXCL10	3627	64
I-TAC	CXCL11, SCYB9B	6373	65
Kallikrein 3	PSA, KLK3	354	66
Leptin	OB	3952	67
LIF	---	3967	68
Lipocalin-2	NGAL, LCN2, Siderocalin	3934	69
MCP-1	CCL2, MCAF	6347	70
MCP-3	CCL7, MARC	6354	71
M-CSF	CSF1	1435	72
MIF	---	4282	73
MIG	CXCL9	4283	74
MIP-1α/MIP-1β	CCL3/CCL4	6348/6351	75
MIP-3α	CCL20, Exodus-1, LARC	6364	76
MIP-3β	CCL19, ELC	6363	77
MMP-9	CLG4B, Gelatinase B	4318	78
Myeloperoxidase	MPO, Lactoperoxidase	4353	79
Osteopontin	OPN	6696	80
PDGF-AA	---	5154	81
PDGF-AB/BB	---	5154/5155	82
Pentraxin-3	PTX3, TSG-14	5806	83
PF4	CXCL4	5196	84
RAGE	---	177	85
RANTES	CCL5	6352	86
RBP4	---	5950	87
Relaxin-2	RLN2, PLXH2	6019	88
Resistin	ADSF, FIZZ3, RETN	56,729	89
SDF-1α	CXCL12, PBSF	6387	90
Serpin E1	PAI-I, PAI-1, Nexin	5054	91
SHBG	ABP	6462	92
ST2	IL-1 R4, IL-1RL1, ST2L	9173	93
TARC	CCL17	6361	94
TFF3	ITF, TFI	7033	95
TfR	CD71, TFR1, TFRC, TRFR	7037	96
TGF-α	TGFA	7039	97
Thrombospondin-1	THBS1, Tsp-1	7057	98
TNF-α	TNFSF1A	7124	99
μPAR	PLAUR	5329	100
VEGF	BEGFA	7422	101
Vitamin D BP	VDB, DBP, VDBP	2638	102

Table S6. Overview of antibodies used for flow cytometry.

Antibody	Origin	Label	Catalogue Number	Provider
IgG_1/IgG_1 isotype simultest control	mouse		349526	Becton Dickinson Biosciences Systems, San Jose, CA, USA
**Mesenchymal stem cell markers**				
CD13	Mouse Anti-human	PE	347406	Becton Dickinson Biosciences Systems, San Jose, CA, USA
CD29	Mouse Anti-human	PE	555443	Becton Dickinson Pharmigen™, San Jose, CA, USA
CD44	Mouse Anti-human	PE	555479	Becton Dickinson Pharmigen™, San Jose, CA, USA
CD54	Mouse Anti-human	PE	347977	Becton Dickinson Biosciences Systems, San Jose, CA, USA
CD71	Mouse Anti-human	PE	555537	Becton Dickinson Pharmigen™, San Jose, CA, USA
CD90	Mouse Anti-human	FITC	555595	Becton Dickinson Pharmigen™, San Jose, CA, USA
CD105	Mouse Anti-human	PE	560839	Becton Dickinson Pharmigen™, San Jose, CA, USA
CD166	Mouse Anti-human	PE	559263	Becton Dickinson PharmigenTM, San Jose, CA, USA
**Hematopoietic stem cell markers**				
CD14	Mouse Anti-human	FITC	345784	Becton Dickinson Biosciences Systems, San Jose, CA, USA
CD34	Mouse Anti-human	FITC	555821	Becton Dickinson PharmigenTM, San Jose, CA, USA
CD45	Mouse Anti-human	FITC	345808	Becton Dickinson Biosciences Systems, San Jose, CA, USA
